# Sigma-1 receptor knockout disturbs gut microbiota, remodels serum metabolome, and exacerbates isoprenaline-induced heart failure

**DOI:** 10.3389/fmicb.2023.1255971

**Published:** 2023-08-31

**Authors:** Jian-Zheng Yang, Kai-Kai Zhang, Hong-Wu Shen, Yi Liu, Xiu-Wen Li, Li-Jian Chen, Jia-Li Liu, Jia-Hao Li, Dong Zhao, Qi Wang, Chu-Song Zhou

**Affiliations:** ^1^Guangzhou Key Laboratory of Forensic Multi-Omics for Precision Identification, School of Forensic Medicine, Southern Medical University, Guangzhou, China; ^2^Key Laboratory of Evidence Science (China University of Political Science and Law), Ministry of Education, Beijing, China; ^3^Security Department, University of Electronic Science and Technology of China, Chengdu, China; ^4^Department of Spine Surgery, Zhujiang Hospital, Southern Medical University, Guangzhou, Guangdong, China

**Keywords:** heart failure, sigma-1 receptor, gut microbiota, untargeted metabolomics, transcriptomics, inflammation

## Abstract

**Introduction:**

Heart failure (HF) is usually the end stage of the continuum of various cardiovascular diseases. However, the mechanism underlying the progression and development of HF remains poorly understood. The sigma-1 receptor (Sigmar1) is a non-opioid transmembrane receptor implicated in many diseases, including HF. However, the role of Sigmar1 in HF has not been fully elucidated.

**Methods:**

In this study, we used isoproterenol (ISO) to induce HF in wild-type (WT) and Sigmar1 knockout (Sigmar1^−/−^) mice. Multi-omic analysis, including microbiomics, metabolomics and transcriptomics, was employed to comprehensively evaluate the role of Sigmar1 in HF.

**Results:**

Compared with the WT-ISO group, Sigmar1^−/−^ aggravated ISO-induced HF, including left ventricular systolic dysfunction and ventricular remodeling. Moreover, Sigmar1^−/−^ exacerbated ISO-induced gut microbiota dysbiosis, which was demonstrated by the lower abundance of probiotics g_Akkermansia and g_norank_f_Muribaculaceae, and higher abundance of pathogenic g_norank_f_Oscillospiraceae and Allobaculum. Furthermore, differential metabolites among WT-Control, WT-ISO and Sigmar^−/−^-ISO groups were mainly enriched in bile secretion, tryptophan metabolism and phenylalanine metabolism, which presented a close association with microbial dysbiosis. Corresponding with the exacerbation of the microbiome, the inflammation-related NOD-like receptor signaling pathway, NF-kappa B signaling pathway and TNF signaling pathway were activated in the heart tissues.

**Conclusion:**

Taken together, this study provides evidence that a Sigmar1 knockout disturbs the gut microbiota and remodels the serum metabolome, which may exacerbate HF by stimulating heart inflammation.

## 1. Introduction

Heart failure (HF) is a common global heart disease with high morbidity and mortality rates, which is a clinical syndrome characterized by abnormal cardiac structure and function (McDonagh et al., 2022). Recent studies have shown that the inflammatory response (Murphy et al., 2020), myocardial interstitial fibrosis (González et al., 2018), apoptosis (Liao et al., 2022) and calcium signaling (Kubalova et al., 2005) promote the development of HF. Despite recent progress in pharmaceutical development, current therapies are inadequate, and outcomes are unsatisfactory (Rosik et al., 2018). Therefore, investigating the underlying molecular mechanisms of HF for developing novel effective therapeutic targets is urgently needed. Sigma-1 receptor (Sigmar1) was originally proposed as an opioid receptor and is expressed widely in the heart, liver, brain and lung (Martin et al., 1976). Subsequent studies have shown that Sigmar1 is an endoplasmic reticulum (ER) transmembrane chaperone protein that is located mainly in the mitochondria-associated ER membrane (Hayashi and Su, 2007) and regulates ER stress, inflammation (Almási et al., 2020), calcium signaling and cell survival (Abdullah et al., 2022). Sigmar1 promotes angiogenesis by activating the JAK2/STAT3 pathway to improve cardiac remodeling and cardiac function in rodent models of HF (Zhao et al., 2022). Haloperidol aggravates transverse aortic constriction-induced ventricular remodeling and HF by inhibiting Sigmar1 (Shinoda et al., 2016). Currently, only a few studies have examined the role of Sigmar1 in the pathogenesis of HF, and relevant multi-omics data are lacking.

In the physiological state, the balanced gut microbiota plays an important role in maintaining the normal cardiovascular system (Jia et al., 2019). In cardiovascular and other diseases, microbial dysbiosis is defined as a change in the microbiome composition (Velmurugan et al., 2020). The gut microbiota affects pathophysiological mechanisms associated with the progression of HF (Trøseid et al., 2020). Gut microbial dysregulation contributes to intestinal barrier disruption, inflammation, oxidative stress and endotoxemia in patients with HF (Yuzefpolskaya et al., 2020). Improving the gut microbiota with a high-fiber diet has been shown to protect hypertensive mice against HF (Marques et al., 2017).

Metabolomics can detect subtle changes in biological pathways, thus providing insights into the mechanisms of various physiological conditions and disease processes (Johnson et al., 2016). Several metabolites, such as short-chain fatty acids, trimethylamine N-oxide and bile acids, are associated with development of HF (Tang et al., 2019). In this report, we performed untargeted metabolomics to study the functional data of Sigmar1 in the pathogenesis of HF. To further evaluate the involvement of Sigmar1 in the pathogenesis of HF, we also performed a transcriptomic analysis of cardiac tissues.

In short, we explored the role of Sigmar1 in an ISO-induced HF model using multi-omics analysis to provide new insights into the pathogenesis of Sigmar1 in the development of HF.

## 2. Materials and methods

### 2.1. Animal models

Male C57BL/6 wild-type (WT) mice (aged 6–8 weeks, certification No. 44826500000653, SPF grade) were purchased from Guangdong Medical Laboratory Animal Center (Guangdong, China). Sigmar1^−/−^ mice on a C57BL/6 background were obtained from Cyagen Biotechnology Co., Ltd. (Guangzhou, China) and bred in the same conditions as WT mice. All mice were maintained in a pathogen-free environment and underwent a 7-day acclimatization period before experiments. Room temperature was maintained at 22°C, and mice were housed in a 12-h light and 12-h dark cycle with food and water *ad libitum*. All experimental procedures involving mice were approved by the Laboratory Animal Ethics Committee of Southern Medical University (Ethical Committee Approval Code: L2022091) and followed the Guide for Care and Use of Laboratory Animals. For HF disease models, isoprenaline hydrochloride (30 mg/kg; Sigma-Aldrich, St Louis, MO, United States, CAS #: 51–30-9) in saline was infused to the mice of WT-ISO and KO-ISO groups with subcutaneous injection daily for 2 weeks as described previously (Feng et al., 2022), while the mice of WT-Control and KO-Control groups were injected with an equal volume of saline. Two mice in the WT-ISO group died on day 13, and two in the KO-ISO group died on days 8 and 13, potentially due to differing tolerances to ISO. To maintain consistency in sample size, we used a total of 32 mice across the four groups (*n* = 8 per group): WT-Control, KO-Control, WT-ISO, and KO-ISO.

### 2.2. Genotype identification

In brief, we excised a small piece of the mouse tail, placed it in 100 μL DNA extraction buffer (Cat #: D7283S, Beyotime, China) and digested this tissue at 55°C for 15 min and 95°C for 5 min. Subsequently, 100 μL termination solution was added to the digestion products and 1 μL was used for amplification after mixing. PCR products were electrophoresed on 2.5% agarose gels and observed under a UV lamp (Cheng et al., 2022). The primer sequences for genotype identification in the experiment are given in Table 1.

**Table 1:** The primers used for genotype identification.

Primers	Name	Sequence (5′–3′)	Size (bp)
Sigmar1^−/−^ PCR	F1	TTCTGTGCTAGCAGACCTAGAAAG	KO: 456
	R1	GCTGTTTAGACACATAAGGAAACGA	
Wild-type PCR	F1	TTCTGTGCTAGCAGACCTAGAAAG	WT: 470
	R2	AGAGAAGACGAAGTTTTGAGTGCC	

### 2.3. Echocardiography

Before transthoracic echocardiography, mice chest hairs were removed with a topical depilatory agent. M-mode echocardiography was performed to evaluate the left ventricular systolic function of WT and Sigmar1^−/−^ mice after saline or ISO administration. Anesthesia was induced by 3% isoflurane and maintained with 1% isoflurane (RWD, Life Science Co., Ltd., China). The mice were then measured with a Vevo 2,100 echocardiography system (FUJIFILM Visualsonics, Toronto, ON, Canada). Images were collected from the left parasternal short axis, and three consecutive cardiac cycles were measured. Measurements of the left ventricular internal dimension in the diastole (LVIDd), left ventricular internal dimension in the systole (LVIDs), left ventricular diastolic volume (LVdVol) and left ventricular systolic volume (LVsVol) were performed. These measurements were used to calculate the left ventricle ejection fraction (LVEF), left ventricle fractional shortening (LVFS) and stroke volume (SV) (Gao et al., 2011).

### 2.4. Histopathological examination

After the above treatment, all mice were sacrificed under 0.3% sodium pentobarbital anesthesia (i.p.) (Chen et al., 2021a), and hearts for tissue fixation were rapidly fixed in 10% formalin for 48 h. Sections of paraffin-embedded tissue were cut at 5-μm thickness and mounted on slides. Hematoxylin and eosin (H&E) staining (Cat #: G1120, Solarbio, Beijing, China), Masson’s trichrome staining (Cat #: G1346, Solarbio) and the wheat germ agglutinin (WGA) assay (Cat #: 25530, AAT Bioquest, United States) were used to evaluate general myocardium morphology, myocardial fibrosis and the cardiomyocyte cross-sectional area. Images of stained slides were observed under a light microscope (Leica DM500, Germany) and a laser confocal microscope. LSM 710; Carl Zeiss Microscopy, Thornwood, NY, United States). For Masson and WGA staining, we selected six random fields of view from three different heart samples in each group.

### 2.5. Biochemical analysis

At 4°C, blood was centrifuged at 3000 g for 15 min to obtain serum samples. The levels of lactate dehydrogenase (LDH) (Cat #: MB-5900A, Jiangsu Meibiao Biotechnology Co., Ltd) and cardiac troponin (cTnT) (Cat #: MB-6288A) in the serum from each sample were detected using ELISA kits, according to the manufacturer’s instructions. Optical densities were measured on a Thermo Scientific Microplate Reader (Thermo Scientific, Waltham, MA, United States) at 450 nm.

### 2.6. Western blot analysis

Heart tissues stored at −80°C were homogenized in lysis buffer (RIPA lysis buffer: protease inhibitors: phosphatase inhibitors = 100: 1: 1) to obtain the total protein. The Pierce™ BCA Protein Assay Kit (Thermo Scientific) was used to measure the protein concentration. Total protein (15 μg, each sample) was resolved by 12% SDS-PAGE and transferred to 0.22-μm PVDF membranes (Merck Millipore, Darmstadt, Germany). Membranes were blocked with 5% skim milk for 2 h at room temperature and incubated with primary antibodies overnight at 4°C. Membranes were then incubated with relevant horseradish peroxidase-labeled secondary antibodies (room temperature, 2 h). Chemiluminescence reagents (Thermo Scientific) were used to visualize the protein bands. Grayscale was calculated by ImageJ (version 1.52i) software with normalization to that of GAPDH. Primary antibodies and dilutions were: Sigmar1 (1,1,000, CST, Cat #: 61994S) and GAPDH (1,1,000, Proteintech, Cat #: 60004-1-Ig).

### 2.7. Real-time quantitative polymerase chain reaction analysis

Total RNA in left ventricular tissue stored at −80°C was extracted using TRIzol reagent (Invitrogen, Waltham, MA, United States), and the RNA concentration was measured by an Agilent 2,100 Bioanalyzer (Agilent Technologies, Santa Clara, CA, United States). Messenger RNA (1 μg, each sample) was then used for complementary DNA synthesis with StarScript III All-in-one RT Mix with gDNA Remover (Cat #: A230-10, GenStar, China) on a reverse transcription system (TransGen Biotech, Beijing, China). Augmentation was performed using 2× RealStar Green Fast Mixture (A301-10, GenStar) on a Roche LightCycler 480 (Roche, Shanghai, China). The relative expression of target genes was calculated by normalization to β-actin using the 2^–△△Ct^ method (Table 2).

**Table 2:** Gene-specific primer sequences for RT-qPCR.

Gene	Forward (5′–3′)	Reverse (5′–3′)
Col1a1	TTCTCCTGGCAAAGACGGAC	CTCAAGGTCACGGTCACGAA
Col3a1	TCAAGCCTGAAGGAAACAGCA	GATGGGTAGTCTCATTGCC
α-SMA	GTCCCAGACATCAGGGAGTAA	TCGGATACTTCAGCGTCAGGA
ANF	CTGGGACCCCTCCGATAGAT	CACTCTGGGCTCCAATCCTG
BNP	GGCTGTAACGCACTGAAGTT	CACTTCAAAGGTGGTCCCAG
β-MHC	TTACTTGCTACCCTCAGGTGG	CTCCTTCTCAGACTTCCGCA
IL-1β	TGCCACCTTTTGACATGATG	AGTGATACTGCCTGCCTGAA
IL-6	GCCTTCTTGGGACTGATGCT	CTGCAAGTGCATCATCGTTGT
TNF-α	ACCACGCTCTTCTGTCTACT	AGGAGGTTGACTTTCTCCTG
β-Actin	GCAGATGTGGATCAGCAAGC	GCAGCTCAGTAACAGTCCGC

### 2.8. 16S ribosomal RNA (rRNA) gene sequencing

According to the manufacturer’s certificate, total bacterial DNA from mouse feces was extracted using the E.Z.N.A.^®^ Soil DNA Kit (Omega Bio-Tek, Norcross, GA, United States). Next, amplification of 16 S rRNA genes was performed using bacterial primers 338 F (5′-ACTCCTACGGGAGGCAGCAG-3′) and 806 R (5′-GGAC TACHVGGGTWTCTAAT-3′) spanning the hypervariable regions of V3-V4. The amplification was carried out on an ABI GeneAmp^®^ 9,700 PCR thermocycler (ABI, CA, United States), followed by purification, pooling and sequencing of amplicons using the Illumina MiSeq platform (Illumina, San Diego, CA, United States). The free online tool, Majorbio I-Sanger Cloud Platform,^1^ was used to analyze the resulting sequence data.

### 2.9. Untargeted metabolomics analysis

Serum samples (100 μL per sample) were extracted using 400 μL methanol:acetonitrile (1:1, v/v). After mixing thoroughly for 30 s and low-temperature ultrasonic extraction for 30 min (5°C, 40 kHz), the sample was statically placed at −20°C for 30 min and centrifuged at 13,000*g* and 4°C for 15 min. The supernatant was carefully transferred to new microtubes and evaporated to dryness under nitrogen, and 100 μL compound solution (acetonitrile:water = 1:1) was added. After re-dissolving, the supernatant was extracted by low-temperature ultrasonic extraction (5°C, 40 kHz) for 5 min, centrifuged at 13,000*g* and 4°C for 5 min and transferred to the injection vial with an inner cannula for analysis. Metabolites of all samples of equal volume were mixed to prepare quality control samples (QC). In instrumental analysis, one QC sample was inserted into every four samples to investigate the repeatability of the whole analytical process. Samples (5 μL per sample) were separated by an HSS T3 chromatographic column (100 × 2.1 mm i.d., 1.8 μm) and detected by mass spectrometry (MS). The positive and negative ion scanning modes were used to collect the sample quality spectrum signal. After MS detection was completed, the raw data of LC/MS were preprocessed by Progenesis QI (Waters Corporation, Milford, United States) software, and a three-dimensional data matrix in CSV format was exported. Concurrently, the metabolites were searched and identified, and the main databases used were HMDB,^2^ Metlin^3^ and Majorbio Database. Differential metabolites with VIP > 1.0 and a *p* value < 0.05 were considered statistically significant. The data after the database search were uploaded to the Majorbio cloud platform for data analysis.^4^

### 2.10. RNA-sequencing analysis

We extracted total RNA from the left ventricle using TRIzol and treated the samples with DNase I to deplete genomic DNA, and then, according to TruSeq™ RNA Sample Preparation Kit from Illumina, enriched mRNA (1 μg per sample) was used to synthesize complementary DNA (cDNA). The remaining steps were the same as those in our previous studies (Chen et al., 2021b; Zhang et al., 2021). Briefly, cDNA was purified by Zymo-Spin IC columns (Zymo Research, CA, United States), and the cDNA fragments were amplified using PCR and sequenced with an Illumina HiSeq™ 2,500 platform (Major Biotechnology Company, Shanghai, China). Differentially expressed genes were identified using log_2_FC (≥ 1) and *p* value (≤ 0.05) filtering. For the presented analysis, gene function, pathway enrichment and heatmap analyses were performed on the Majorbio I-Sanger Cloud Platform. And all heatmaps and correlation analyses were performed in OmicStudio.^5^

### 2.11. Statistical analysis

All quantitative results were expressed as mean ± SEM. To determine whether two groups differed statistically, we used the two-tailed unpaired Student’s *t*-test or Welch’s *t*-test (unequal variances), whereas differences across multiple groups were performed with One-way ANOVA with Tukey’s multiple comparisons test or Brown-Forsythe and Welch ANOVA tests with Games-Howell’s multiple comparisons test (unequal variances). *p* < 0.05 was considered statistically significant. Data analysis was performed using GraphPad Prism 8.0.2.

## 3. Results

### 3.1. Chronic ISO induced heart failure and transcriptomic alterations in WT mice

The establishment of an HF model was confirmed by performing transthoracic echocardiography after the last ISO injection. As shown in Supplementary Figures S1A–D, mice injected with ISO showed a significant decrease in cardiac function as indicated by reductions in LVEF, LVFS and SV when compared with the corresponding values of the WT-Control group. In addition, the left ventricular inner diameter and volume at end-diastole did not change significantly in the ISO-treated mice when compared with the corresponding values in the control group, whereas the inner diameter and volume at end-systole increased, suggesting that ISO induced systolic cardiac dysfunction (Supplementary Figures S1F,G). For histomorphology, HE and Masson staining showed inflammatory cell infiltration and collagen deposition in the extracellular matrix of the myocardium in the WT-ISO group (Supplementary Figures S1E,H). In comparison to the WT-Control group, relative fiber area and the mRNA levels of the fibrosis markers, α-SMA (α-smooth muscle actin), Col1a1 (collagen, type I, alpha 1) and Col3a1 (collagen, type III, alpha 1), increased in the WT-ISO group (Supplementary Figures S1I,J). WGA staining in the WT-ISO group showed a larger cross-sectional area of the ventricular myocardium (Supplementary Figures S1K,L). Moreover, treatment with ISO increased the heart weight to tibia length (HW/TL), heart weight to body weight (HW/BW) and the mRNA levels of cardiac failure and hypertrophy markers ANF (atrial natriuretic factor) BNP (brain natriuretic peptide), and hypertrophy marker β-MHC (β-myosin heavy chain) (Supplementary Figures S1M–O). In the serum of ISO-treated mice, LDH and cTnT levels (biomarkers of myocardial injury) were higher than those in the control group (Supplementary Figures S1P,Q). These results showed that ISO induced ventricular remodeling and myocardial injury.

**Figure 1:**
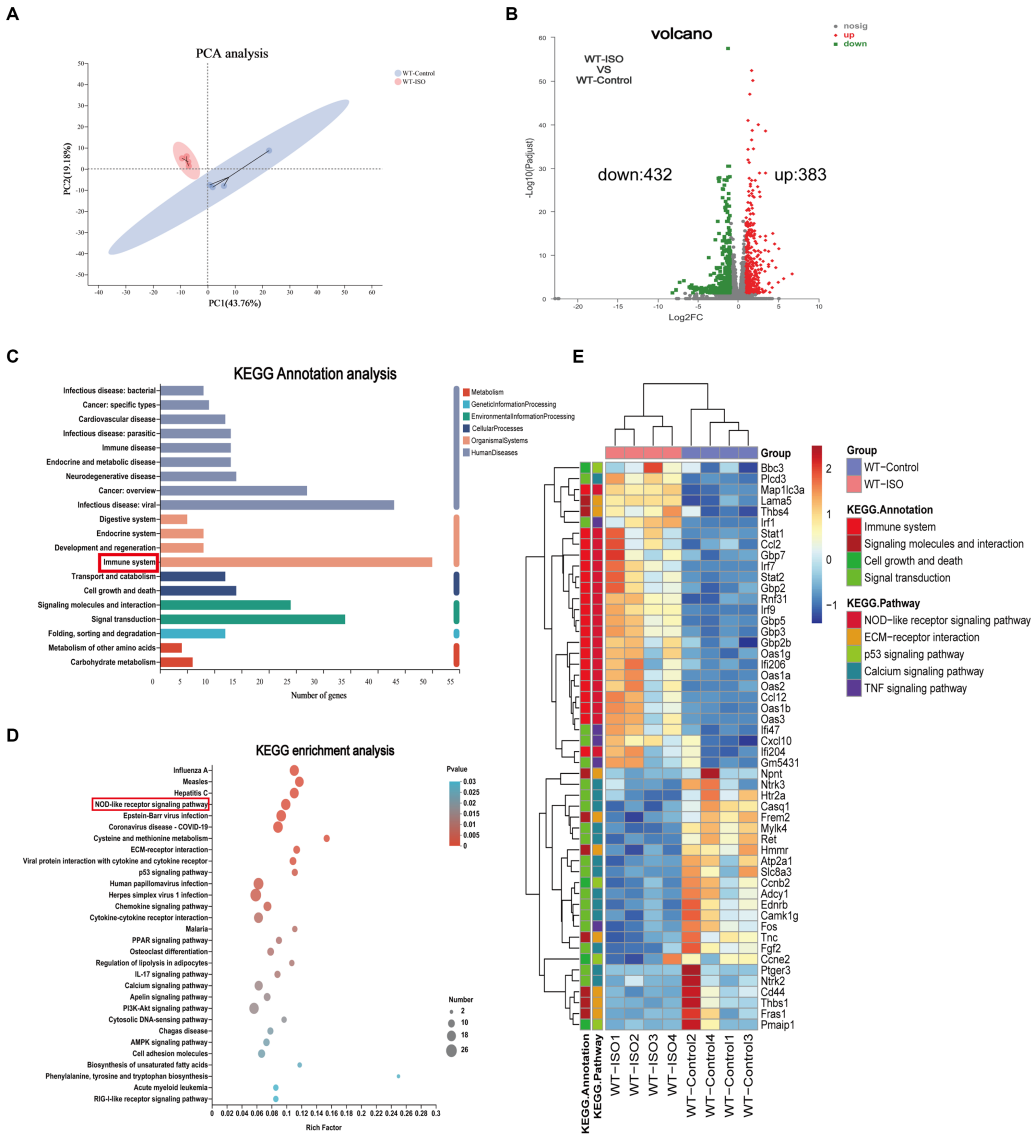
Transcriptome analysis between WT-Control and WT-ISO group. **(A)** Principal component analysis (PCA). *n* = 4 mice per group. **(B)** Volcano plot of RNA-seq indicating the DEGs in heart samples from WT mice with or without ISO treatment. **(C)** Kyoto Encyclopedia of Genes and Genomes (KEGG) annotation analysis. **(D)** The KEGG enrichment analysis revealed the top 30 pathways. **(E)** The heatmap shows the DEGs of five enriched pathways in WT-Control and WT-ISO groups.

The mechanism of ISO-induced HF was further explored by performing RNA-sequencing of left ventricular tissues from WT mice with or without ISO treatment. Principal component analysis (PCA) showed distinct clustering of gene profiles between the two groups (Figure 1A). We then identified 815 differentially expressed genes (DEGs) through log_2_FC (≥ 1) and the *p* value (≤ 0.05). Compared with the WT-Control group, ISO treatment upregulated 383 genes and downregulated 432 genes (Figure 1B), and these DEGs were mainly annotated to the immune system (Figure 1C). Furthermore, KEGG enrichment analysis gave the top 30 significantly enriched pathways, and we found that the NOD-like receptor signaling pathway, which is related to the immune system, was enriched. Notably, the calcium signaling pathway, which is associated with cardiovascular diseases, was also enriched (Figure 1D). The heatmap between the two groups showed that inflammation-related genes were upregulated significantly, whereas calcium signaling-related genes were downregulated significantly in the WT-ISO group (Figure 1E). Therefore, the results suggested that ISO treatment altered the heart transcriptome, which activated inflammatory pathways and altered calcium signaling.

### 3.2. Chronic ISO induced gut microbiota dysbiosis and metabolite changes in WT mice

**Figure 2:**
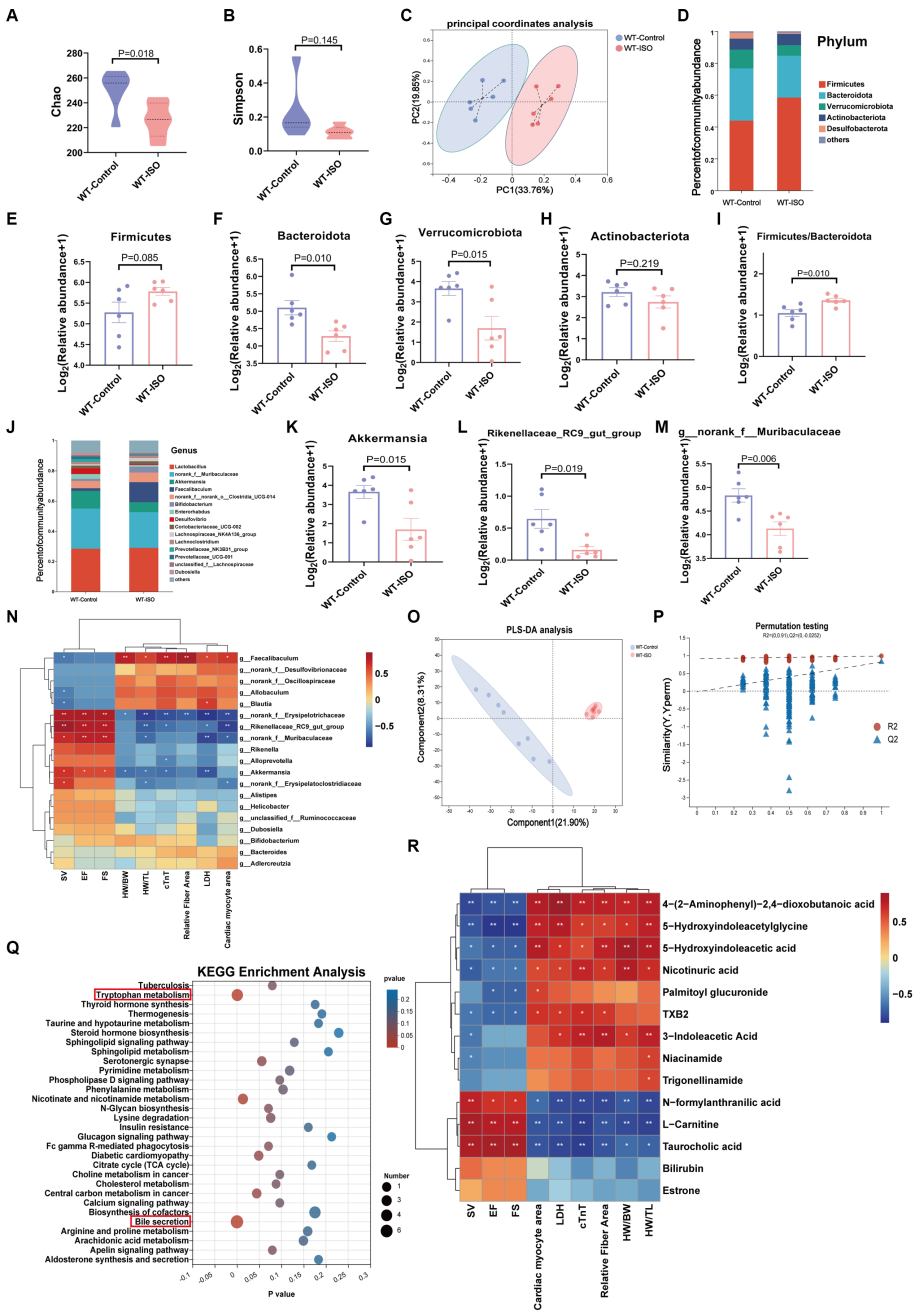
Gut microbiota and metabolic alteration between the WT-Control and WT-ISO groups. **(A,B)** The Chao and Simpson index were examined to assess alpha diversity in indicated groups. **(C)** Bray_curtis principal coordinates analysis (PCoA) was used to indicate the β-diversity of gut microbiota. **(D)** A stacked bar graph of both groups showed differential bacteria at the phylum level. **(E–H)** Analysis of the relative abundance of the four major bacterial groups at the phylum level. **(I)** The increased Firmicutes/Bacteroidota ratio indicated ISO-induced bacterial dysbiosis. **(J)** Relative abundance of gut microbiota genera in each group. **(K–M)** The abundance of representative bacteria genera. *n* = 6 mice per group. **(N)** Spearman correlation analysis for 19 altered genera and nine cardiac-related measures. **(O,P)** The PLS-DA analysis and the corresponding coefficient of loading plots indicated significant metabolite changes between the WT-Control and WT-ISO groups. *n* = 8 mice per group. **(Q)** KEGG enrichment analysis of 182 differential metabolites in both groups. **(R)** Spearman correlation analysis between 14 metabolites from the top three enriched pathways and nine cardiac-related measures.

To explore the potential relationship between the gut microbiota and metabolites in mediating ISO-induced HF, we performed 16S rRNA gene sequencing and untargeted metabolomics analysis. The Chao index showed that the bacterial species richness in the WT-ISO group was lower than that in the WT-Control group, but the Simpson index did not differ significantly between the two groups (Figures 2A,B), which suggested that the administration of ISO decreased the bacterial species richness and did not altered the bacterial diversity. Principal coordinates analysis (PCoA) showed distinct clustering of microbiota composition in mice treated with saline or ISO (Figure 2C). At the phylum level, Firmicutes and Bacteroidota were dominant in the two groups, with lower abundance of Bacteroidota and Verrucomicrobia, and a higher abundance of Firmicutes/Bacteroidota in the WT-ISO group than in the WT-Control group (Figures 2D–I). At the genus level, changes in gut microbiota composition were observed, as shown in the stacked bar chart (Figure 2J). For six representative taxa, the abundance of Akkermansia, Rikenellaceae_RC9_gut_group and g_norank_f_Muribaculaceae was significantly lower in the WT-ISO group than in the WT-Control group (Figures 2K–M), whereas the abundance of Alistipes, g_norank_f_Oscillospiraceae and Allobaculum showed changing trends but no significant differences (Supplementary Figures S2A–C). Furthermore, correlation analysis based on the Spearman correlation coefficient was performed to explore the correlation between gut microbiota with significantly altered genus-level abundance and cardiotoxicity markers. The analysis illustrated that Akkermansia, Rikenellaceae_RC9_gut_group and g_norank_f_Muribaculaceae positively correlated with the cardiac function indices (EF, FS, and SV) but negatively correlated with the measures of cardiac injury and remodeling (Figure 2N).

In the untargeted metabolic analysis, PLS-DA analysis showed that the two groups of metabolites were clustered separately, and permutation testing (*R*^2^ = 0.987, *Q*^2^ = 0.819) showed that the PLS-DA models were robust (Figures 2O,P). A total of 186 metabolites in the serum between the WT-Control and WT-ISO groups were detected through screening conditions of VIP > 1 and *p* value < 0.05, and KEGG functional pathways were primarily enriched in amino acid metabolism, lipid metabolism and the digestive system (Supplementary Figures S2D,E). KEGG enrichment analysis showed augmentation primarily in bile secretion, tryptophan metabolism and nicotinate and nicotinamide metabolism. Notably, similar to the transcriptome, the calcium signaling pathway was also enriched (Figure 2Q). Correlation analysis between differential metabolites from the top three pathways and cardiotoxicity markers showed that niacin and nicotinamide metabolites (nicotinuric acid, niacinamide) and tryptophan metabolites [4-(2-aminophenyl)-2,4-dioxobutanoic acid, 5-hydroxyindoleacetic acid, 3-indoleacetic acid and 5-hydroxyindoleacetylglycine] were negatively correlated with the cardiac function index. Taurocholic acid, which is related to bile secretion, was positively correlated with the cardiac function index (Figure 2R). We further explored the functional correlation between the disturbed gut microbes and major altered metabolites, which revealed that Rikenellaceae_RC9_gut_group, g_norank_f_Muribaculaceae and Akkermansia were positively correlated with taurocholic acid and negatively correlated with tryptophan metabolites (Supplementary Figure S2F).

### 3.3. Knockout of Sigmar1 caused a decrease in cardiac function at baseline and exacerbated ISO-induced left ventricular systolic dysfunction and ventricular remodeling

**Figure 3:**
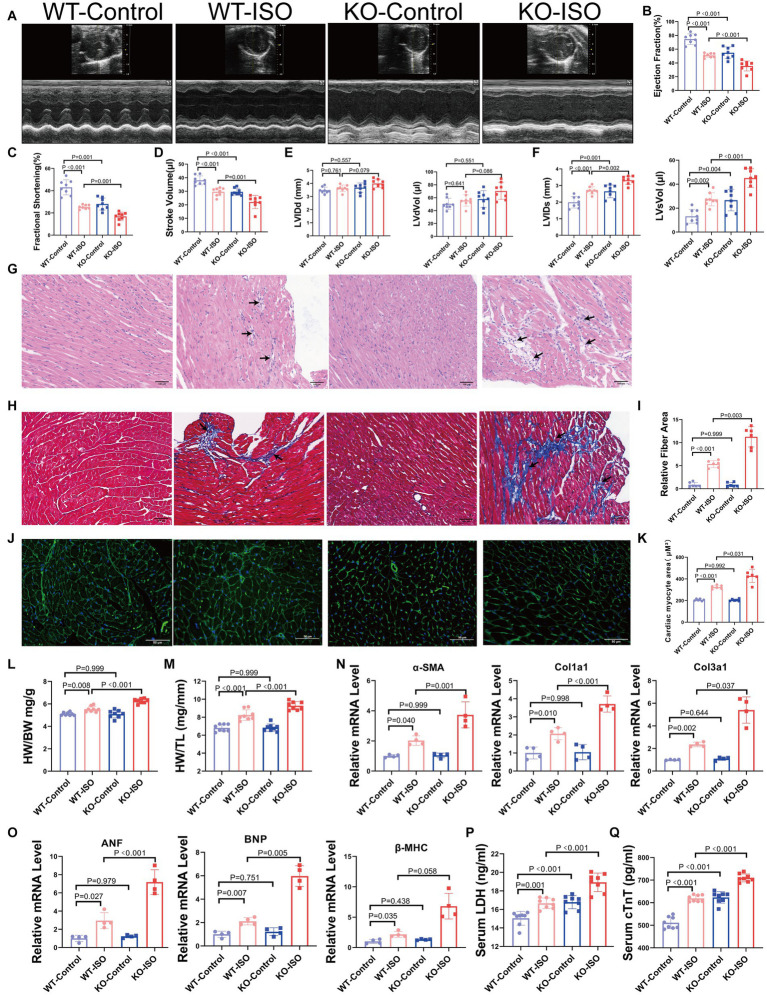
Sigmar1 knockout aggravated ISO-induced HF. **(A)** Representative echocardiographic images in each group. **(B–D)** Measurements of EF, FS, and SV from M-mode images for each group. **(E,F)** Analysis of LVIDd, LVdVol, LVIDs, and LVsVol among the four groups. *n* = 8 mice per group. **(G)** Representative HE staining of heart sections (scale bars, 100 μm). **(H,I)** Masson staining and quantitative analysis of fibrotic (blue) areas (scale bars, 100 μm). *n* = 3 mice per group. **(J,K)** WGA staining and quantitative analysis of cardiomyocyte areas (scale bars, 50 μm). *n* = 3 mice per group. **(L,M)** HW/BW and HW/TL were measured in each group to assess myocardial hypertrophy. *n* = 8 mice per group. **(N)** Relative mRNA levels of cardiac fibrosis marker genes (α-SMA, Col1a1, Col3a1) for each group. **(O)** The mRNA levels of heart failure markers (ANF and BNP) and the cardiac hypertrophy marker (β-MHC) among the four groups. *n* = 4 mice per group. **(P,Q)** Serum levels of myocardial injury markers LDH and cTnT in the four groups. *n* = 8 mice per group.

We found that Sigmar1 expression was downregulated in the left ventricle of ISO-treated mice by western blot and RT-qPCR analyses (Supplementary Figures S3A,B). To explore the role of Sigmar1 in heart function, WT and Sigmar1^−/−^ mice were treated with ISO (30 mg/kg/day) subcutaneously for 14 days, and the knockout of Sigmar1 was confirmed by PCR (Supplementary Figures S3C,D). Cardiac function measurements in WT and Sigmar1^−/−^ mice were performed by echocardiographic. At baseline, Sigmar1^−/−^ mice showed a decline in EF, FS and SV and an increase in LVIDs and LVsVol when compared with the corresponding values in the WT-Control group, which indicated that the mice in the KO-Control group developed systolic cardiac insufficiency at 9 weeks of age. Moreover, Sigmar1^−/−^ mice treated with ISO showed lower EF, FS and SV than the WT-ISO group and higher LVIDs and LVsVol than the WT-ISO group. These results suggest that the Sigmar1 knockout aggravated ISO-induced ventricular systolic dysfunction (Figures 3A–F).

HE staining and Masson staining showed that compared with the WT-ISO group, mice in the KO-ISO group showed more inflammatory cell infiltration and collagen deposition in the extracellular matrix of the myocardium (Figures 3G–I). WGA staining revealed a significant increase in the cross-sectional area of the ventricular myocardium in ISO-treated mice, and Sigmar1 knockout aggravated the myocardial hypertrophy (Figures 3J,K). Additionally, Sigmar1 knockout further increased the ISO-induced elevation of HW/BW and HW/TL (Figures 3L,M). RT-qPCR showed that the relative mRNA levels of α-SMA, Col1a1, Col3a1, ANF, BNP and β-MHC in the KO-ISO group were higher than those in the WT-ISO group (Figures 3N,O). These results suggested that Sigmar1 knockout aggravated ISO-induced ventricular remodeling. In addition, the levels of LDH and cTnT in the serum of KO-Control group were higher than those in the serum of WT-Control group. Compared with the WT-ISO group, the KO-ISO group exhibited higher levels of LDH and cTnT (Figures 3P,Q). These results showed that Sigmar1 knockout induced myocardial damage at baseline and that Sigmar1 knockout aggravated ISO-induced myocardial damage.

### 3.4. Microbiome, metabolome and transcriptome alterations between the KO-Control and WT-Control groups

**Figure 4:**
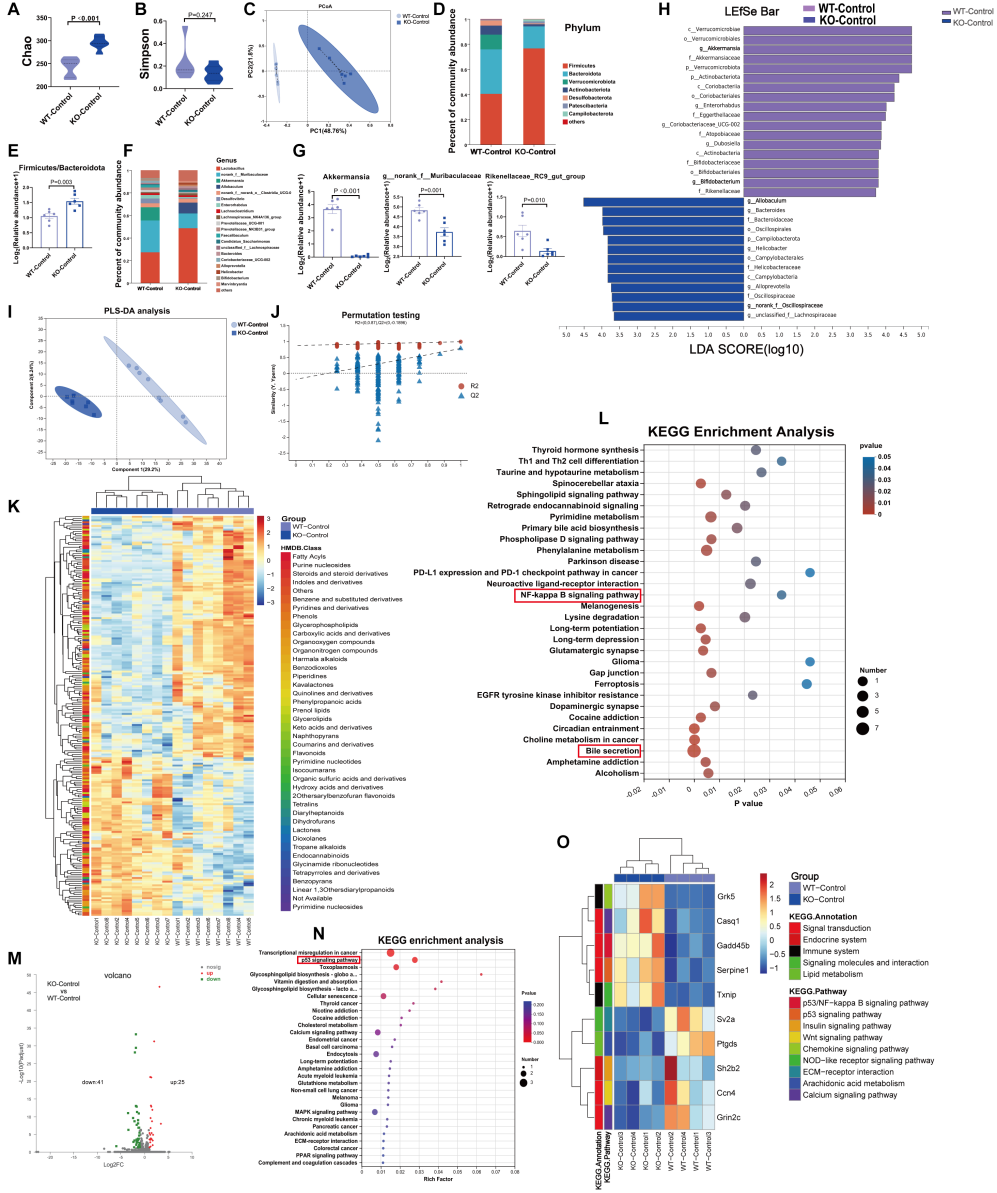
At baseline, Sigmar1^−/−^ mice had different gut microbiota, metabolites and transcriptomes compared with WT mice. **(A,B)** The Chao and Simpson diversity index was examined by 16S-rRNA sequencing. **(C)** PCoA analysis showed that the gut microbial composition clustered separately in both groups. **(D,E)** Analysis of species composition at the phylum level and the ratio of F/B showed gut microbiota dysbiosis between WT-Con and KO-Con groups. **(F,G)** Analysis of species composition at the genus level and the relative abundance of representative genus-level bacteria. **(H)** Linear discriminant analysis (LDA) histograms reflected significant differences in gut microbial abundance in the WT-Con and KO-Con groups. *n* = 6 mice per group. **(I,J)** PLS-DA analysis and the corresponding coefficient of loading plots indicated significant metabolite changes between the WT-Con and KO-Con groups. **(K)** A total of 217 differential metabolites are shown in the heatmap between the WT-Con and KO-Con groups. **(L)** KEGG enrichment analysis of differential metabolites in both groups. *n* = 8 mice per group. **(M,N)** Volcano plot showing 66 DEGs, and KEGG showing the top 30 enriched pathways. **(O)** The heatmap shows the gene levels of partial pathways in WT-Con and KO-Con groups. *n* = 4 mice per group. Con: Control.

At baseline, Sigmar1^−/−^ mice showed a decrease in cardiac function and an increase in serum LDH and cTnT levels when compared with those in WT mice. We performed 16S rRNA gene sequencing, untargeted metabolomics and RNA-sequencing analysis to investigate the underlying mechanisms. The Chao index showed that the bacterial species richness in the KO-Control group was higher than that in the WT-Control group, but the Simpson index, which represents bacterial diversity, did not differ significantly between the two groups (Figures 4A,B). PCoA analysis showed that the microbiota composition was clearly separated between the two groups (Figure 4C). At the phylum level, the abundance of Bacteroidota, Verrucomicrobiota and Actinobacteriota was lower in the KO-Control group (Supplementary Figure S4A). The abundance of Firmicutes and Firmicutes/Bacteroidota in the KO-Control group was higher than in the WT-Control group (Figures 4D,E). At the genus level, the abundance of Rikenellaceae_RC9_gut_group, g_norank_f_Muribaculaceae, Akkermansia and Alistipes was significantly lower in the KO-Control group (Figures 4F,G), and the abundance of Allobaculum and g_norank_f_Oscillospiraceae was significantly higher in the KO-Control group (Supplementary Figure S4B). The cladogram showed that the compositions of gut microbiota communities varied significantly between the two groups (Supplementary Figure S4C). Linear discriminant analysis (LDA ≥ 3.5) showed that Akkermansia was most enriched in the WT-Control group, and Allobaculum was most enriched in the KO-Control group (Figure 4H). In summary, the gut microbiota results showed that Sigmar1 knockout caused significant dysbiosis at baseline, which was mainly manifested as a decrease in Akkermansia and an increase in Allobaculum.

The metabolite compositions of the two groups clustered separately, as shown in Figures 4I,J (*R*^2^ = 0.99, *Q*^2^ = 0.777). The heatmap showed 217 differential metabolites between the WT-Control and KO-Control groups (VIP ≥ 1 and *p* ≤ 0.05) (Figure 4K), with KEGG functional pathways mainly enriched in amino acid and lipid metabolism and the digestive system (Supplementary Figure S4D). KEGG enrichment analysis revealed enrichment mainly in bile secretion. Moreover, we noticed that the NF-kappa B signaling pathway was enriched (Figure 4L). Furthermore, RNA-sequencing analysis revealed 25 upregulated genes and 41 downregulated genes in the KO-Control group (log_2_FC ≥ 1) and *p* value ≤ 0.05 (Figure 4M), with KEGG annotation analysis mainly enriched in signal transduction and the immune system (Supplementary Figure S4E). The enriched pathways by DEGs also included important pathways in developing HF, such as the p53 signaling and calcium signaling pathways. Subsequent heatmaps showed that Gadd45b, which has been shown to reduce cardiac function and induce cardiac fibrosis, was upregulated significantly in the KO-Control group (Figures 4N,O). Combining the metabolic and transcriptomic data, we hypothesized that knockout of Sigmar1 at baseline causes an enhanced tendency toward inflammation, apoptosis and fibrosis.

### 3.5. Knockout of Sigmar1 exacerbated ISO-induced gut microbiota dysbiosis

**Figure 5:**
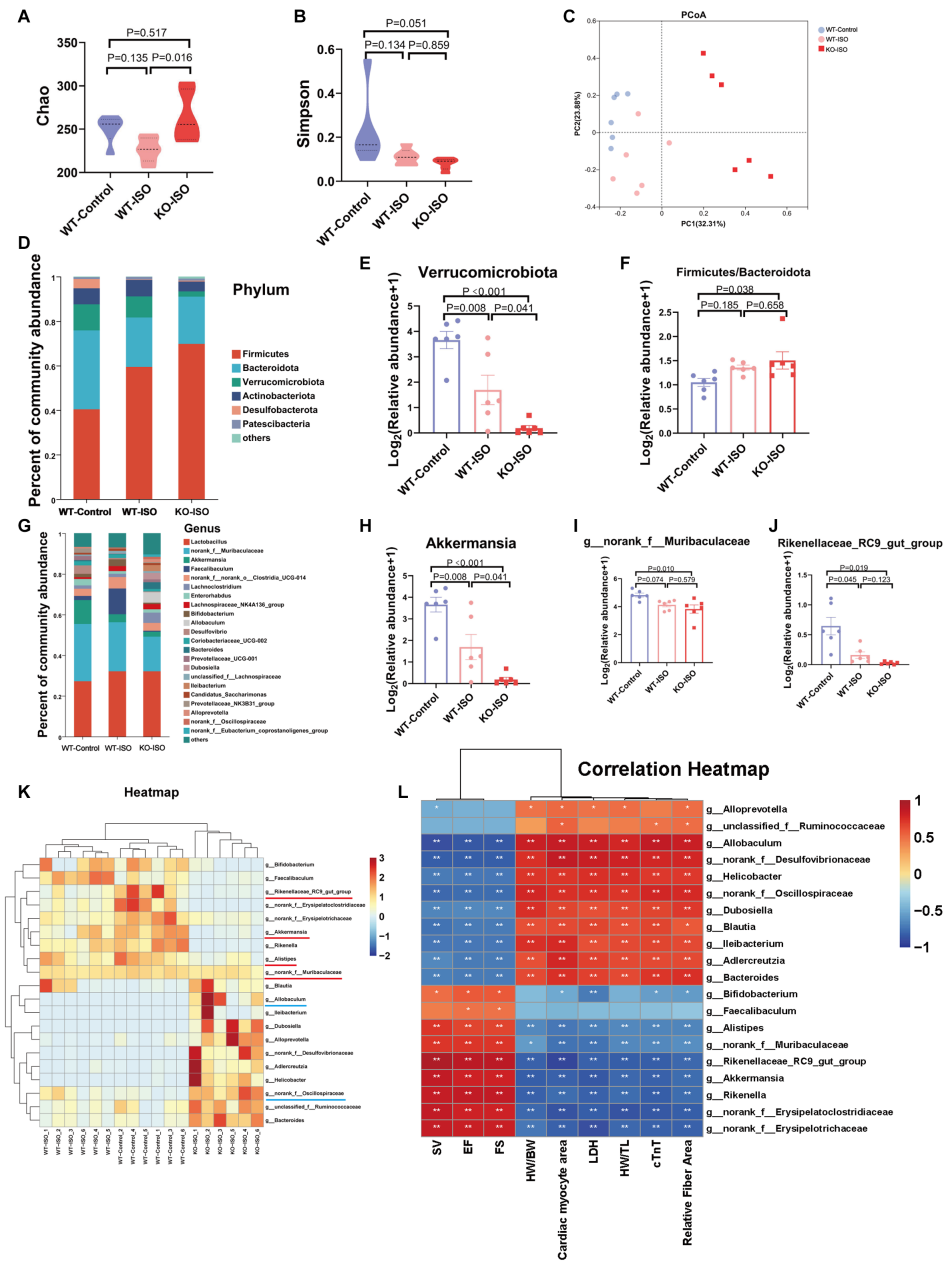
Sigmar1 knockout further aggravated ISO-induced gut microbiota dysbiosis. **(A)** The Chao index showed that the species richness of the microbiota was abnormally increased in the KO-ISO group. **(B)** The Simpson index showed that the diversity of species was further increased in the KO-ISO group when compared with the WT-ISO group. **(C)** PCoA (Bray_curtis) showed that the microbial composition of the KO-ISO group was clearly separated from that of the other two groups. **(D)** The stacked bar chart shows differences in species composition at phylum level. **(E,F)** The relative abundance of Verrucomicrobiota and Firmicutes/Bacteroidota. *n* = 6 mice per group. **(G)** The stacked bar chart shows differences in species composition at genus level. **(H–J)** Relative abundance analysis of a representative gut bacterial genus in the three groups. *n* = 6 mice per group. **(K)** Heatmap analysis for 20 altered genera in the three groups. **(L)** Spearman correlation analysis for 20 altered genera and nine cardiac-related indices.

To investigate the composition of gut microbiota co-regulated by ISO and Sigmar1, we compared WT-Control, WT-ISO and KO-ISO groups. Compared with the WT-ISO group, the KO-ISO group exhibited a higher Chao index and lower Simpson index, which showed the species richness of the microbiota was increased in the KO-ISO group and the diversity of species was further increased in the KO-ISO group (Figures 5A,B). Bray_curtis PCoA revealed that the KO-ISO group showed a marked difference in microbial composition when compared with the microbial compositions of the WT-Control and WT-ISO groups (Figure 5C). Differences in gut microbiota composition among the three groups at the phylum and genus levels were examined using Sankey diagram (Supplementary Figure S5A) and stacked bar charts (Figures 5D,G). Specifically, the abundance of Verrucomicrobiota and Actinobacteriota was lower in the KO-ISO group at the phylum level, and the abundance of Firmicutes and Firmicutes/Bacteroidota was higher in the KO-ISO group than in the WT-ISO group (Figures 5E,F and Supplementary Figures S5B–D). The relative abundance of 20 altered genera among the three groups is presented in the heatmap (Figure 5K). Specially, the abundance of Akkermansia, g_norank_f_Muribaculaceae, Rikenellaceae_RC9_gut_group and Alistipes were further reduced in the KO-ISO group when compared with the WT-ISO group, g_norank_f_Oscillospiraceae and Allobaculum increased significantly in the KO-ISO group (Figures 5H–J and Supplementary Figures S5E–G). These results suggested that Sigmar1 knockout aggravated gut microbiota dysbiosis induced by ISO. Furthermore, the correlation heatmap based on the spearman analysis illustrated the 20 altered genera strongly correlated with the cardiac function indices and the measures of cardiac injury and remodeling (Figure 5L).

### 3.6. Metabolome and transcriptome alterations among the WT-Control, WT-ISO and KO-ISO groups

**Figure 6:**
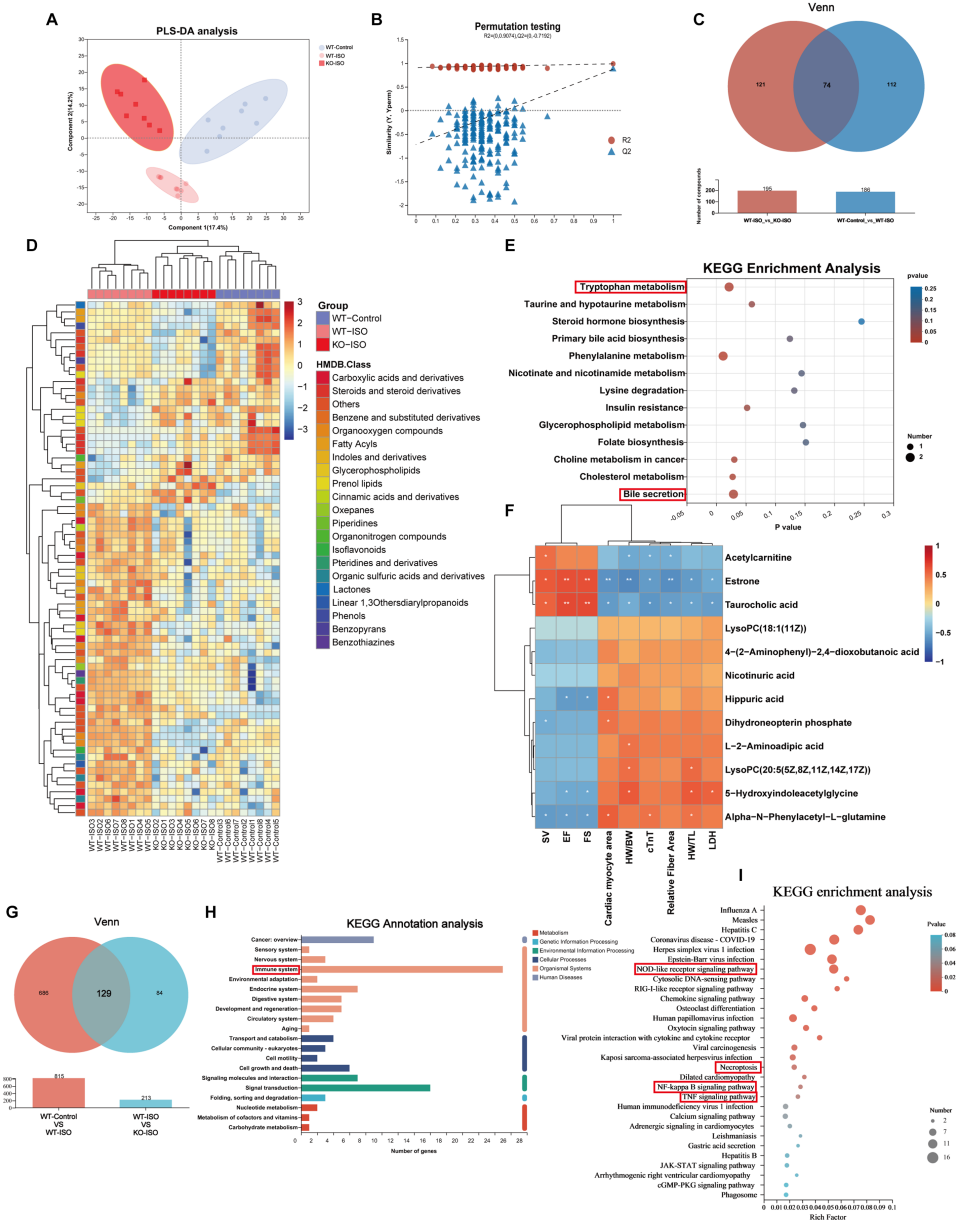
Metabolome and transcriptome alterations among the WT-Control, WT-ISO and KO-ISO groups. **(A,B)** The PLS-DA models indicated significant metabolic variations among the WT-Con, WT-ISO and KO-ISO groups. *n* = 8 mice per group. **(C)** Venn diagram of differential metabolites between WT-Con and WT-ISO mice and between WT-ISO and KO-ISO mice. **(D)** The heatmap shows 74 differential metabolites and HMDB classification. **(E)** KEGG enrichment analysis of 74 differential metabolites. **(F)** Spearman correlation analysis for 12 altered metabolites from the first six enriched pathways and nine cardiac-related measures. **(G)** Venn diagram of DEGs between WT-Con and WT-ISO mice and between WT-ISO and KO-ISO mice. **(H,I)** KEGG annotation analysis and enrichment analysis of 74 DEGs.

Untargeted metabolomics and transcriptomics were performed to investigate the alterations in metabolism and transcriptomes co-regulated by ISO and Sigmar1. PLS-DA analysis and a permutation test (*R*^2^ = 0.986, *Q*^2^ = 0.871) showed a distinct separation among the three groups (Figures 6A,B). The Venn diagram showed that there were 186 ISO-regulated differential metabolites between WT-Control and WT-ISO, 195 Sigmar1-regulated differential metabolites between WT-ISO and KO-ISO, and 74 metabolites that were co-regulated by ISO and Sigmar1 (Figure 6C). Subsequently, a heatmap was used to show the expression levels and HMDB compound classification of 74 differential metabolites (Figure 6D), with KEGG enrichment analysis mainly enriched in bile secretion, tryptophan metabolism, phenylalanine metabolism, cholesterol metabolism and insulin resistance (Figure 6E). We performed a correlation analysis between metabolites enriched in these major pathways and the cardiac index, and found that estrone and taurocholic acid were significantly positively correlated with cardiac function, whereas 5-hydroxyindoleacetylglycine, alpha-N-phenylacetyl-L-glutamine and hippuric acid were significantly negatively correlated with cardiac function (Figure 6F).

Venn diagram of the transcriptome showed that there were 815 ISO-regulated DEGs between WT-Control and WT-ISO, 213 Sigmar1-regulated DEGs between WT-ISO and KO-ISO, and 129 DEGs that were co-regulated by ISO and Sigmar1 (Figure 6G). KEGG annotation analysis of the 129 DEGs showed that the immune system was enriched significantly, and the NOD-like receptor signaling pathway, NF-kappaB signaling pathway and TNF signaling pathway were enriched in KEGG enrichment analysis (Figures 6H,I). These results suggest that Sigmar1^−/−^ may exacerbate the development of HF by activating inflammation.

To further verify the involvement of an inflammatory response in the exacerbation of ISO-induced HF caused by Sigmar1 deletion, we evaluated the mRNA levels of inflammatory factors in the heart tissue of the four mouse groups. Our results indicated that relative mRNA levels of IL-1β, IL-6, and TNF-α in the KO-ISO group were significantly higher than those in the WT-ISO group (Supplementary Figures S6A–C). This supports the notion that Sigmar1 deletion intensifies ISO-induced upregulation of cardiac inflammatory factors. Correlation analysis based on the Spearman correlation coefficient was conducted to explore the relationship between heart failure and inflammatory factors. The analysis revealed that IL-1β, IL-6, and TNF-α negatively correlated with cardiac function indices (EF, FS, and SV) but positively correlated with measures of cardiac injury and remodeling (Supplementary Figure S6D).

## 4. Discussion

In this study, we found that ISO-induced HF was accompanied by gut microbiota dysbiosis, alteration of serum metabolites and upregulation of inflammatory genes, whereas Sigmar1 knockout aggravated ISO-induced cardiac dysfunction, ventricular remodeling and increased the levels of cTnT and LDH in serum. Multi-omics analysis found that Sigmar1 knockout further aggravated ISO-induced gut microbiota dysbiosis and activated inflammation-related pathways. In addition, we found that compared with WT mice, sigmar1^−/−^ mice exhibited reduced cardiac function and higher serum cTnT and LDH levels at baseline. The probiotic g__norank_f_Muribaculaceae and Akkermansia and the anti-inflammatory metabolite taurocholic acid were reduced significantly in sigmar1^−/−^ mice, while inflammatory genes were upregulated significantly in sigmar1^−/−^ mouse heart tissues.

ISO-induced HF is an animal model that comprehensively recapitulates the major aspects of human HF, such as ventricular dysfunction, myocardial fibrosis and myocardial hypertrophy (Oudit et al., 2003; Zhou et al., 2018). In our experiment, ISO treatment induced a decrease in the expression level of Sigmar1. Sigmar1 has been demonstrated to play a protective role in the cardiovascular system (Bhuiyan and Fukunaga, 2011). The Sigmar1 agonist SA4503 can improve cardiac hypertrophy and dysfunction in mice with HF (Hirano et al., 2014). In this study, 2-month-old Sigmar1^−/−^ mice showed reduced myocardial systolic function with elevated myocardial injury indicators cTnT and LDH, but ventricular remodeling was not observed at baseline. The serum cTnT level is raised in patients with HF and correlates negatively with cardiac function (Wang et al., 2021). Serums from patients with chronic cardiac failure have been shown to induce a higher level of LDH and apoptosis (Mammi et al., 2011). In the context of ISO induced HF, Sigmar1 knockout aggravated ISO-induced ventricular remodeling and further reduced cardiac function when compared with WT-ISO mice. The levels of cTnT and LDH in serum were higher in the KO-ISO group than in the WT-ISO group. These results suggest that Sigmar1 plays an essential role in cardiac function and structure under physiological and pathological conditions.

A previous study has revealed changes in the structure and function of gut microbial diversity in an ISO-induced rat HF model (Zheng et al., 2019). Dysbiosis of the gut microbiota, intestinal hypoperfusion and hyperemia may alter intestinal permeability and cause microbial translocation, which may cause low-grade systemic inflammation and, in turn, contribute to the progression of HF (Dicken and Cleland, 2014; Lewis and Taylor, 2020). Akkermansia is associated with structural and functional changes in HF progression (Gutiérrez-Calabrés et al., 2020). *Akkermansia muciniphila*, a sentinel of intestinal permeability, is important for maintaining intestinal barrier integrity (Ouyang et al., 2020) and reduces inflammation and prevents heart disease in animal models (Bavineni et al., 2019). In this study, the abundance of Akkermansia, *Akkermansia muciniphila*, g__norank_f_Muribaculaceae, Rikenellaceae_RC9_gut_group and Alistipes were distinctly reduced in the KO-Control group compared with the WT-Control group. In addition, ISO reduced the abundance of Akkermansia, *Akkermansia muciniphila*, g__norank_f_Muribaculaceae, Alistipes and Rikenellaceae_RC9_gut_group, whereas knockout of Sigmar1 exacerbated ISO-induced microbial dysbiosis. g__norank_f_Muribaculaceae has been shown to maintain intestinal homeostasis and reduce inflammation (He et al., 2022). Fecal microbiota transplantation from normal mice can increase the abundance of Alloprevotella and Rikenellaceae_RC9_gut_group to reduce intestinal damage and improve cardiac function (An et al., 2021). The abundance of Alistipes in HF mice is reduced and correlates positively with cardiac function (Guo et al., 2021). In addition, we found increases in the abundance of Allobaculum and g_norank_f_Oscillospiraceae in Sigmar1^−/−^ mice, regardless of whether ISO was administrated. DOX has been shown to increase the abundance of Escherichia Shigella, Dubosiella and Allobaculum and enhanced the inflammatory state in mice to induce cardiotoxicity (Lin et al., 2021). The abundance of norank_f_Oscillospiraceae was correlated positively with neuroinflammation (IL-1β, IL-6) (Wu Y. et al., 2022). In short, Sigmar1 deficiency caused an increase in the abundance of potentially pathogenic bacteria, and the abundance of beneficial bacteria that maintain intestinal barrier homeostasis and anti-inflammation decreased sharply, which may contribute to systemic inflammation and thus promote HF progression. Fecal microbiota transplantation (FMT) can reduce myocardial injury by restoring gut microbiota composition (Hu et al., 2019). Therefore, methods to use these gut microbiota as therapeutic agents, such as probiotics or FMT, require further investigation.

Metabolomics can detect subtle changes in biological pathways to gain insight into the mechanisms of various physiological conditions and disease processes (Johnson et al., 2016). We found that the differential metabolites between WT-Control and KO-Control groups were mainly enriched in bile secretion, and the level of the main metabolite taurocholic acid decreased significantly in Sigmar1^−/−^ mice. Taurocholic acid, a naturally occurring component of animal bile acids, has been shown to be effective in treating various inflammatory diseases (Wang et al., 2013). In the Sigmar1^−/−^ mice, we observed a reduction in taurocholic acid, which could represent an impairment in anti-inflammatory capability. Notably, the NF-kappa B signaling pathway was also enriched. Thus, Sigmar1 deficiency decreased the level of taurocholic acid, leading to the activation of inflammatory pathways at baseline. The differential metabolites among WT-Control, WT-ISO and KO-ISO groups were also enriched in bile secretion, and taurocholic acid correlated positively with probiotics Akkermansia and g__norank_f_Muribaculaceae, and correlated negatively with potentially pathogenic bacteria Allobaculum and g_norank_f_Oscillospiraceae. In addition, we found significant enrichment in the tryptophan metabolism pathway. Prior research has suggested that cardiac pressure overload can induce gut dysbiosis, contributing to heart remodelling. It has been suggested that tryptophan metabolites could potentially contribute to the prevention and treatment of adverse cardiac remodelling and systolic dysfunction in heart failure (Carrillo-Salinas et al., 2020). Consequently, we propose that both the bile secretion pathway (particularly the role of taurocholic acid) and the tryptophan metabolism pathway are implicated in how Sigmar1 knockout affects the progression of heart failure.

Transcriptome analysis was employed to further explore the underlying molecular mechanisms, and the results showed that the significant DEGs between the WT-Control and KO-Control groups were mainly enriched in the p53 signaling pathway. Specifically, Gadd45b and Serpine1 were upregulated in Sigmar1^−/−^ mice. Increased expression of circNlgn reduces cardiac function and promotes fibrosis by upregulating Gadd45b, Sema4C and RAD50 in the heart (Xu et al., 2022). Overexpression of Gadd45b induces the expression of proinflammatory cytokines significantly (IL-1β, IL-8 and TNF-α), which plays an important role in the innate immune response (Bai et al., 2018). An inflammatory response promotes ventricular remodeling and contributes to the development of HF (Grosman-Rimon et al., 2020). Serpine1, a pro-fibrotic gene involved in ECM regulation in cardiomyocytes (Tsoutsman et al., 2013; Tsai et al., 2021), is important in the pathogenesis of HF and may be used for the diagnosis and treatment of HF (Yu et al., 2016). DEGs among WT-Control, WT-ISO and KO-ISO groups were mainly enriched in the NOD-like receptor signaling pathway, NF-kappa B signaling pathway, necroptosis and TNF signaling pathway. Apoptosis, inflammation and fibrosis of the heart lead to left ventricular hypertrophy and cardiac dysfunction (García et al., 2017). MiR-30a-5P promotes HF by activating the NF-kappa B/NOD-like receptor 3 signaling pathways (Wu Y. X. et al., 2022). Necroptosis and apoptosis are closely associated with HF (Zhang et al., 2016). When NOD-like receptors detect tissue damage or microbial infection, they activate IRE1α to recruit TRAF2 to the ER membrane and initiate an inflammatory response through NF-κB (Keestra-Gounder et al., 2016). ER stress promotes apoptosis, cardiac hypertrophy and HF (Yao et al., 2017). However, stimulation of the Sigma-1 receptor can prevent cardiac hypertrophy and fibrosis by alleviating the IRE1 pathway (Qu et al., 2021). Fluvoxamine, an agonist of Sigmar1, reduces ER stress by inducing Sigmar1 (Omi et al., 2014). These findings suggest that Sigmar1 knockout may be involved in the pathogenesis of HF by promoting inflammation and apoptosis, leading to ventricular remodeling. Targeting Sigmar1 represents a potential therapeutic approach, and further mechanistic verification is needed.

## 5. Conclusion

In summary, our findings show that Sigmar1 knockout altered the gut microbiota and serum metabolites and exacerbated ISO-induced HF. This study provides valuable insights into the potential of Sigmar1 as a therapeutic target for the treatment of HF.

## Data availability statement

The datasets presented in this study are deposited in the NCBI Sequence Read Archive (SRA) https://www.ncbi.nlm.nih.gov/sra under accession numbers PRJNA914875 and PRJNA916456.

## Ethics statement

All experimental procedures involving mice were approved by the Laboratory Animal Ethics Committee of Southern Medical University (L2022091) and followed the Guide for Care and Use of Laboratory Animals. The study was conducted in accordance with the local legislation and institutional requirements.

## Author contributions

J-ZY and K-KZ: Investigation, Methodology, Writing – original draft. H-WS: Data curation, Software. YL: Formal analysis, Validation. X-WL: Formal analysis, Validation. L-JC: Project administration. J-LL: Resources. J-HL: Resources. DZ: Funding acquisition, Supervision. QW: Conceptualization, Writing – review & editing. C-SZ: Funding acquisition, Supervision.

## Funding

This work was supported by the General Program of National Natural Science Foundation of China (grant number 81971796, China).

## Conflict of interest

The authors declare that the research was conducted in the absence of any commercial or financial relationships that could be construed as a potential conflict of interest.

## Publisher’s note

All claims expressed in this article are solely those of the authors and do not necessarily represent those of their affiliated organizations, or those of the publisher, the editors and the reviewers. Any product that may be evaluated in this article, or claim that may be made by its manufacturer, is not guaranteed or endorsed by the publisher.

## Figures and Tables

**Table:** 

Sigmar1	sigma-1 receptor
Sigmar1^−/−^	Sigmar1 knockout
ER	endoplasmic reticulum
LVIDd	left ventricular internal dimension in the diastole
LVIDs	left ventricular internal dimension in the systole
LVdVol	left ventricular diastolic volume
LVsVol	left ventricular systolic volume
LVEF	left ventricle ejection fraction
LVFS	left ventricle fractional shortening
SV	stroke volume
H&E	Hematoxylin and eosin
WGA	wheat germ agglutinin
LDH	lactate dehydrogenase
cTnT	cardiac troponin
QC	quality control
α-SMA	α-smooth muscle actin
Col1a1	collagen, type I, alpha 1
Col3a1	collagen, type III, alpha 1
ANF	atrial natriuretic factor
BNP	brain natriuretic peptide
β-MHC	β-myosin heavy chain
PCA	Principal component analysis
DEGs	differentially expressed genes
PCoA	Principal coordinates analysis
IL-1β	interleukin-1 beta
IL-6	interleukin-6
TNF-α	tumor necrosis factor-alpha

## Footnotes

^1^http://www.cloud.majorbio.com

^2^http://www.hmdb.ca/

^3^https://metlin.scripps.edu/

^4^https://cloud.majorbio.com

^5^https://www.omicstudio.cn/tool

## Supplementary material

SUPPLEMENTARY FIGURE S1 ISO-induced ventricular remodeling and systolic dysfunction. **(A)** Representative echocardiogram image for each group. **(B-D)** Results of ejection fraction (EF), fractional shortening (FS) and stroke volume (SV). *n* = 8 mice per group. **(E)** Representative HE staining (scale bars, 100 μm). **(F, G)** Results of LVIDd, LVdVol, LVIDs and LVsVol for each group. *n* = 8 mice per group. **(H)** Representative Masson staining (scale bars, 100 μm). **(I)** Quantitative analysis of fiber areas (blue). *n* = 3 mice per group. **(J)** RT-qPCR showing the mRNA levels of α-SMA, Col1a1 and Col3a1 in heart tissue. *n* = 4 mice per group. **(K)** Representative WGA staining (scale bars, 50 μm). **(L)** Quantitative analysis of the cardiomyocyte areas. n = 3 mice per group. **(M, N)** Analysis of HW/TL and HW/BW. *n* = 8 mice per group. **(O)** mRNA expression levels of ANF, BNP and β-MHC. *n* = 4 mice per group. **(P, Q)** Serum LDH and cTnT levels in the WT-Control and WT-ISO groups. *n* = 8 mice per group.

SUPPLEMENTARY FIGURE S2 Alterations in gut microbiota and metabolites in WT-Control and WT-ISO group. Relative abundance of **(A)** Alistipes **(B)** g_norank_f__Oscillospiraceae **(C)** Allobaculum at genus level. *n* = 6 mice per group. **(D)** Heatmap showed 182 differential metabolites and HMDB compound classification. **(E)** KEGG functional pathways analysis. **(F)** Spearman correlation between 18 altered genus and 14 altered metabolites.

SUPPLEMENTARY FIGURE S3 ISO treatment decreased the expression of sigmar1; electrophoresis results of amplified mouse tail DNA. **(A)** Western blotting analysis showed significantly lower expression level of sigmar1 in WT-ISO group. *n* = 3 mice per group. **(B)** RT-qPCR showing significantly lower mRNA levels of sigmar1. *n* = 4 mice per group. **(C)** The F1/R1/R2 primer was used for PCR amplification, and the wild-type allele (sigmar1^+/+^) showed one band with a size of 470 bp, whereas homozygotes (sigmar1^-/-^) showed one band with a size of 456 bp. Heterozygotes (sigmar1^+/-^) showed two bands with sizes of 456 bp and 470 bp; **(D)** The F1/R2 primer was used for PCR, and wild-type mice showed one band with a size of 470 bp, whereas sigmar1^-/-^ mice showed no band. *n* = 3 mice per group. **(E)** Body weight change indicated groups. *n* = 8 mice per group.

SUPPLEMENTARY FIGURE S4 Microbiome, metabolome, and transcriptome alterations between the KO-Control and WT-Control groups. Relative abundance of **(A)** Firmicutes, Bacteroidota, Verrucomicrobiota and Actinobacteriota at phylum level. Relative abundance of **(B)** Allobaculum, Alistipes and g_norank_f__Oscillospiraceae at genus level. *n* = 6 mice per group. **(C)** Comparison of taxonomic abundances using LEfSe. **(D)** KEGG functional pathways analysis in metabolomics. **(E)** KEGG annotation analysis in transcriptomics.

SUPPLEMENTARY FIGURE S5 Microbiome and metabolome alterations among WT-Control, WT-ISO and KO-ISO groups. **(A)** Sankey diagram was performed to analysis of species composition at the phylum and genus level. Relative abundance of **(B)** Firmicutes, **(C)** Bacteroidota and **(D)** Actinobacteriota at phylum level. Relative bundance of **(E)** Alistipes **(F)** g_norank_f__Oscillospiraceae and **(G)** Allobaculum at genus level. *n* = 6 mice per group.

SUPPLEMENTARY FIGURE S6 Sigmar1^-/-^ exacerbated ISO-induced elevated expression of cardiac inflammatory factors. The mRNA levels of **(A)** IL-1β, **(B)** IL-6 and **(C)** TNF-α among the four groups. **(D)** Spearman correlation analysis for 3 inflammatory factors and 9 cardiac-related indices.
