# Supplementary figures and images for: Sigma-1 receptor knockout disturbs gut microbiota, remodels serum metabolome, and exacerbates isoprenaline-induced heart failure
Source: Front Microbiol. 2023 Aug 31;14:1255971. doi: 10.3389/fmicb.2023.1255971 (PMC10501138; doi:10.3389/fmicb.2023.1255971)

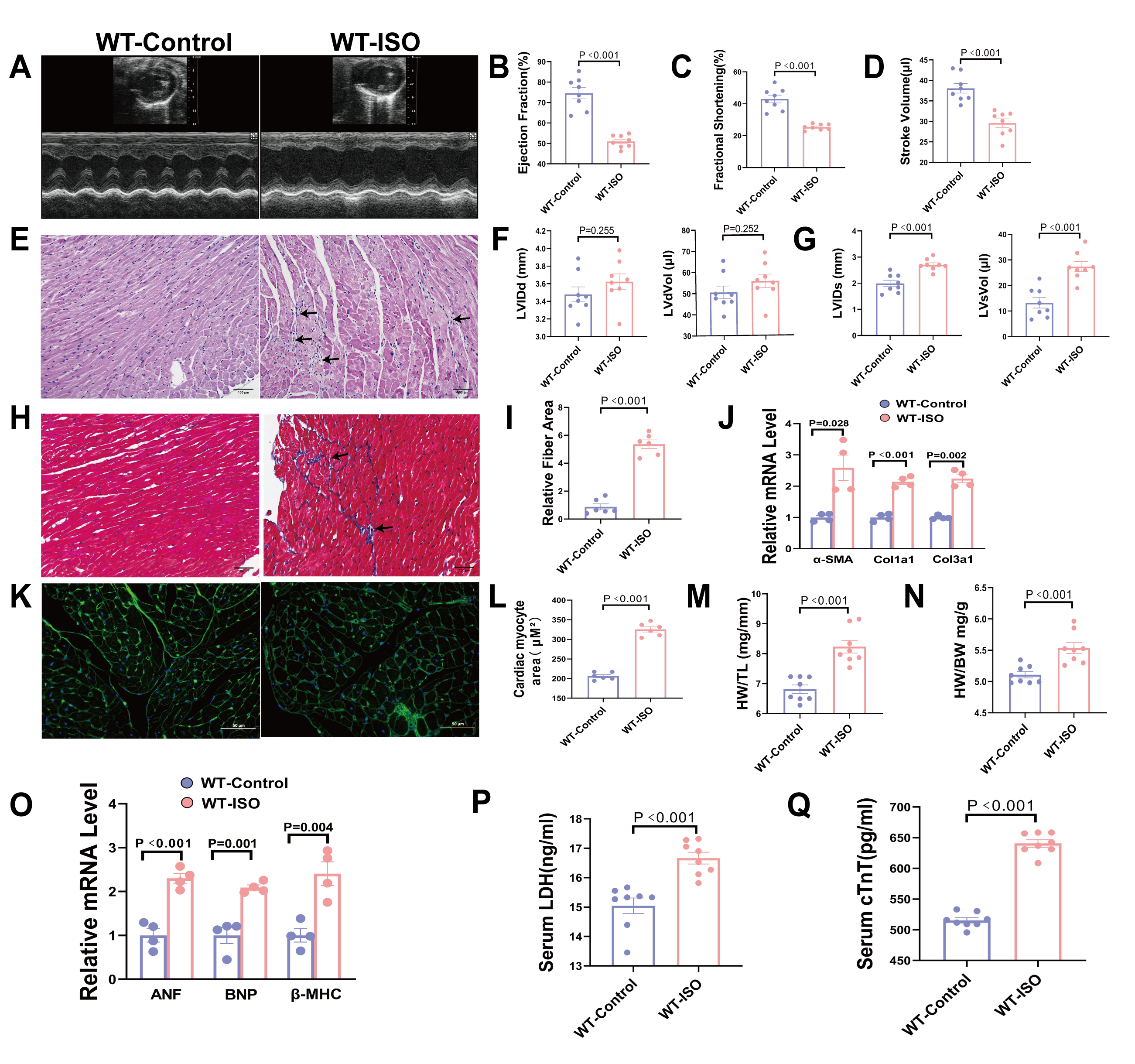

Supplement: SUPPLEMENTARY FIGURE S1 — ISO-induced ventricular remodeling and systolic dysfunction. (A) Representative echocardiogram image for each group. (B-D) Results of ejection fraction (EF), fractional shortening (FS) and stroke volume (SV). n = 8 mice per group. (E) Representative HE staining (scale bars, 100 μm). (F, G) Results of LVIDd, LVdVol, LVIDs and LVsVol for each group. n = 8 mice per group. (H) Representative Masson staining (scale bars, 100 μm). (I) Quantitative analysis of fiber areas (blue). n = 3 mice per group. (J) RT-qPCR showing the mRNA levels of α-SMA, Col1a1 and Col3a1 in heart tissue. n = 4 mice per group. (K) Representative WGA staining (scale bars, 50 μm). (L) Quantitative analysis of the cardiomyocyte areas. n = 3 mice per group. (M, N) Analysis of HW/TL and HW/BW. n = 8 mice per group. (O) mRNA expression levels of ANF, BNP and β-MHC. n = 4 mice per group. (P, Q) Serum LDH and cTnT levels in the WT-Control and WT-ISO groups. n = 8 mice per group. [file Image_1.TIF]

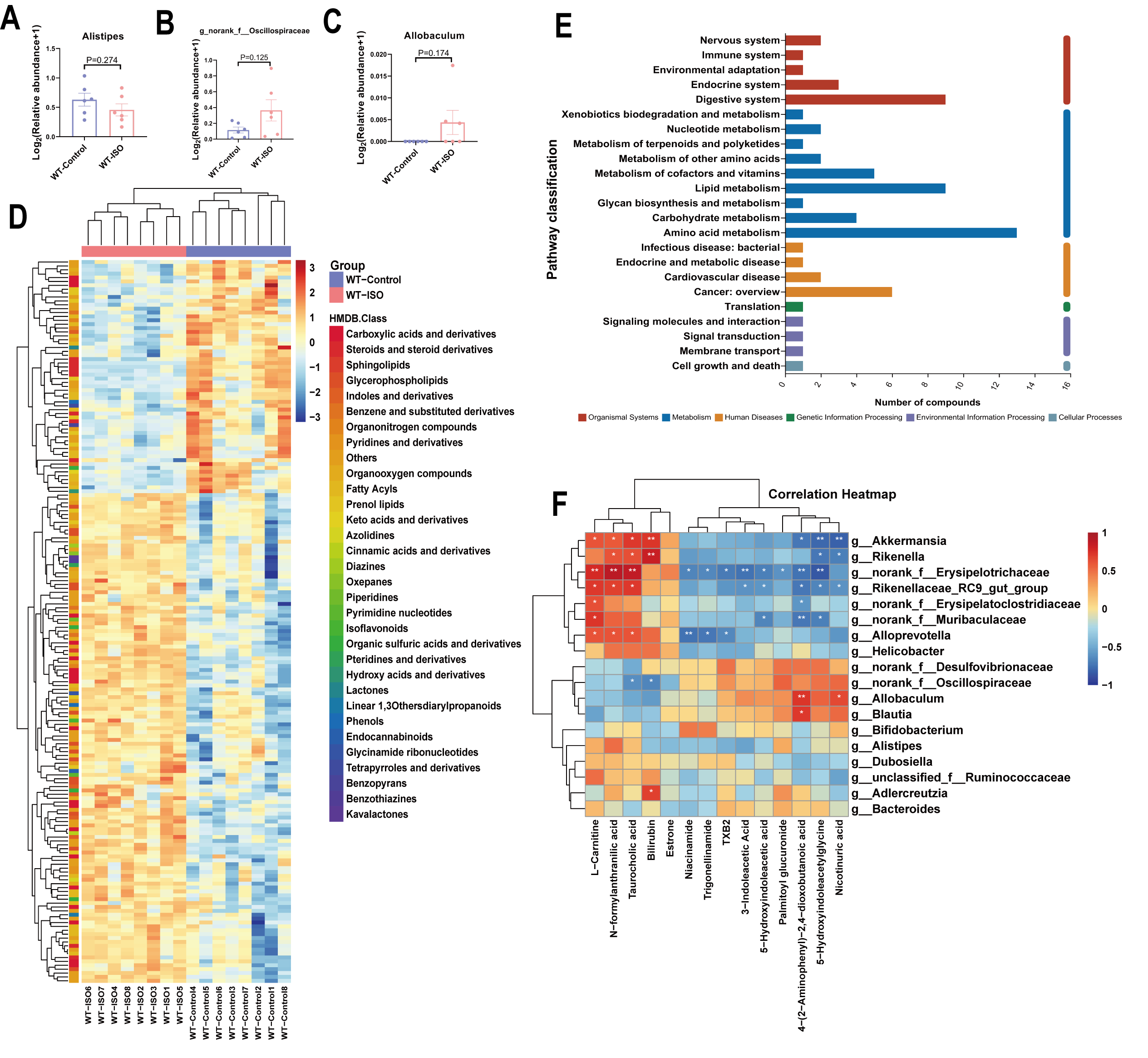

Supplement: SUPPLEMENTARY FIGURE S2 — Alterations in gut microbiota and metabolites in WT-Control and WT-ISO group. Relative abundance of (A) Alistipes (B) g_norank_f__Oscillospiraceae (C) Allobaculum at genus level. n = 6 mice per group. (D) Heatmap showed 182 differential metabolites and HMDB compound classification. (E) KEGG functional pathways analysis. (F) Spearman correlation between 18 altered genus and 14 altered metabolites. [file Image_2.TIF]

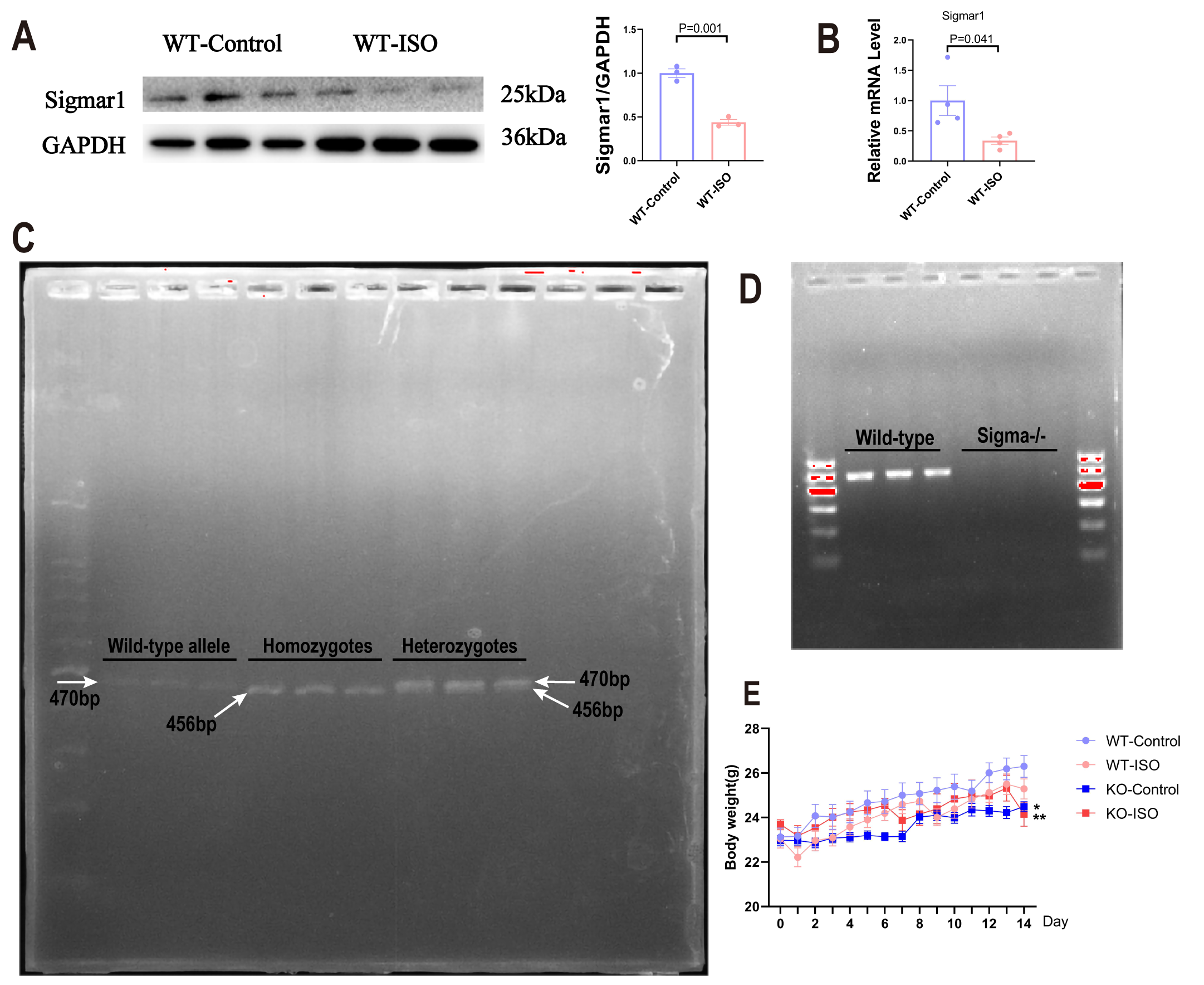

Supplement: SUPPLEMENTARY FIGURE S3 — ISO treatment decreased the expression of sigmar1; electrophoresis results of amplified mouse tail DNA. (A) Western blotting analysis showed significantly lower expression level of sigmar1 in WT-ISO group. n = 3 mice per group. (B) RT-qPCR showing significantly lower mRNA levels of sigmar1. n = 4 mice per group. (C) The F1/R1/R2 primer was used for PCR amplification, and the wild-type allele (sigmar1+/+) showed one band with a size of 470 bp, whereas homozygotes (sigmar1-/-) showed one band with a size of 456 bp. Heterozygotes (sigmar1+/-) showed two bands with sizes of 456 bp and 470 bp; (D) The F1/R2 primer was used for PCR, and wild-type mice showed one band with a size of 470 bp, whereas sigmar1-/- mice showed no band. n = 3 mice per group. (E) Body weight change indicated groups. n = 8 mice per group. [file Image_3.TIF]

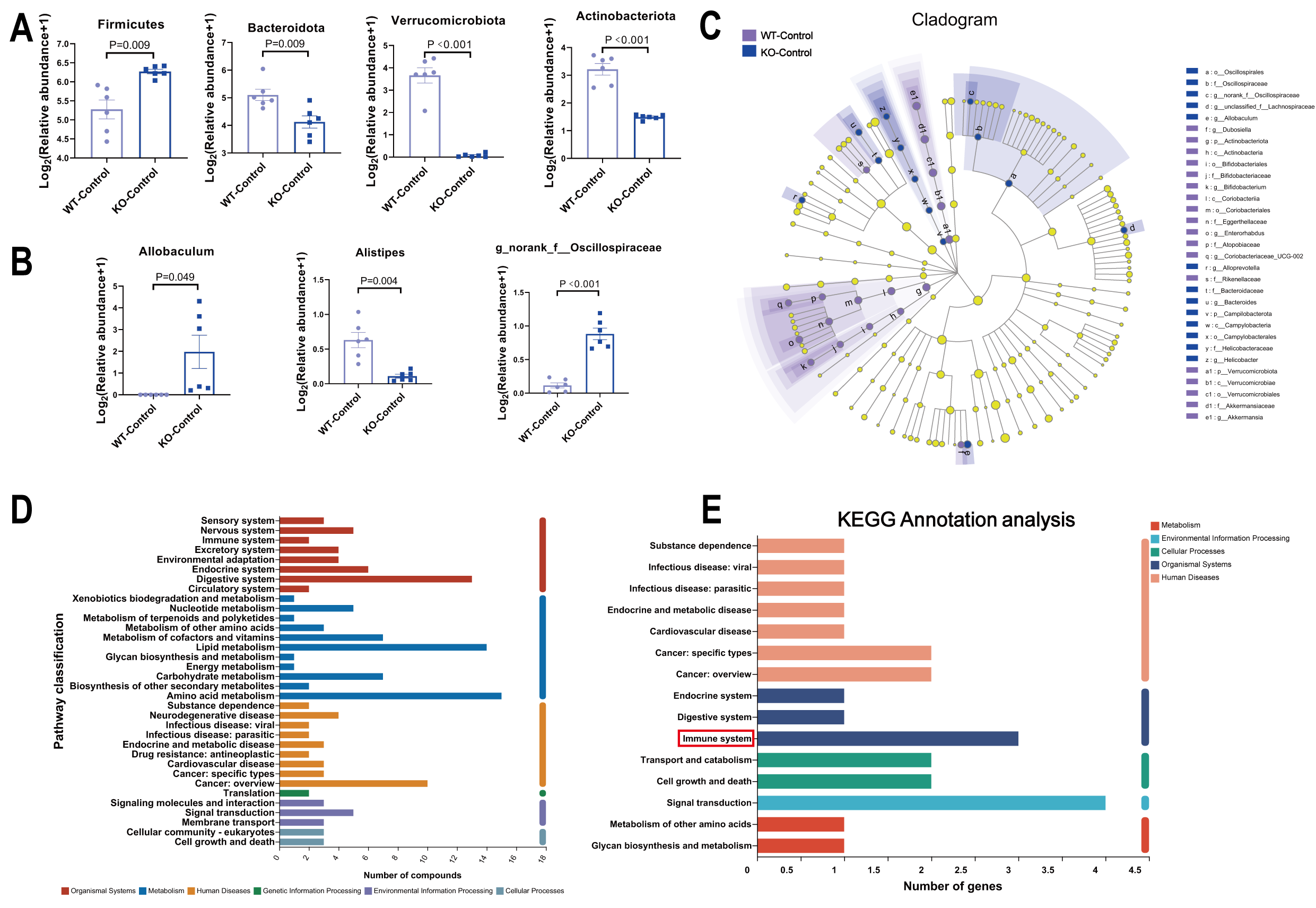

Supplement: SUPPLEMENTARY FIGURE S4 — Microbiome, metabolome, and transcriptome alterations between the KO-Control and WT-Control groups. Relative abundance of (A) Firmicutes, Bacteroidota, Verrucomicrobiota and Actinobacteriota at phylum level. Relative abundance of (B) Allobaculum, Alistipes and g_norank_f__Oscillospiraceae at genus level. n = 6 mice per group. (C) Comparison of taxonomic abundances using LEfSe. (D) KEGG functional pathways analysis in metabolomics. (E) KEGG annotation analysis in transcriptomics. [file Image_4.TIF]

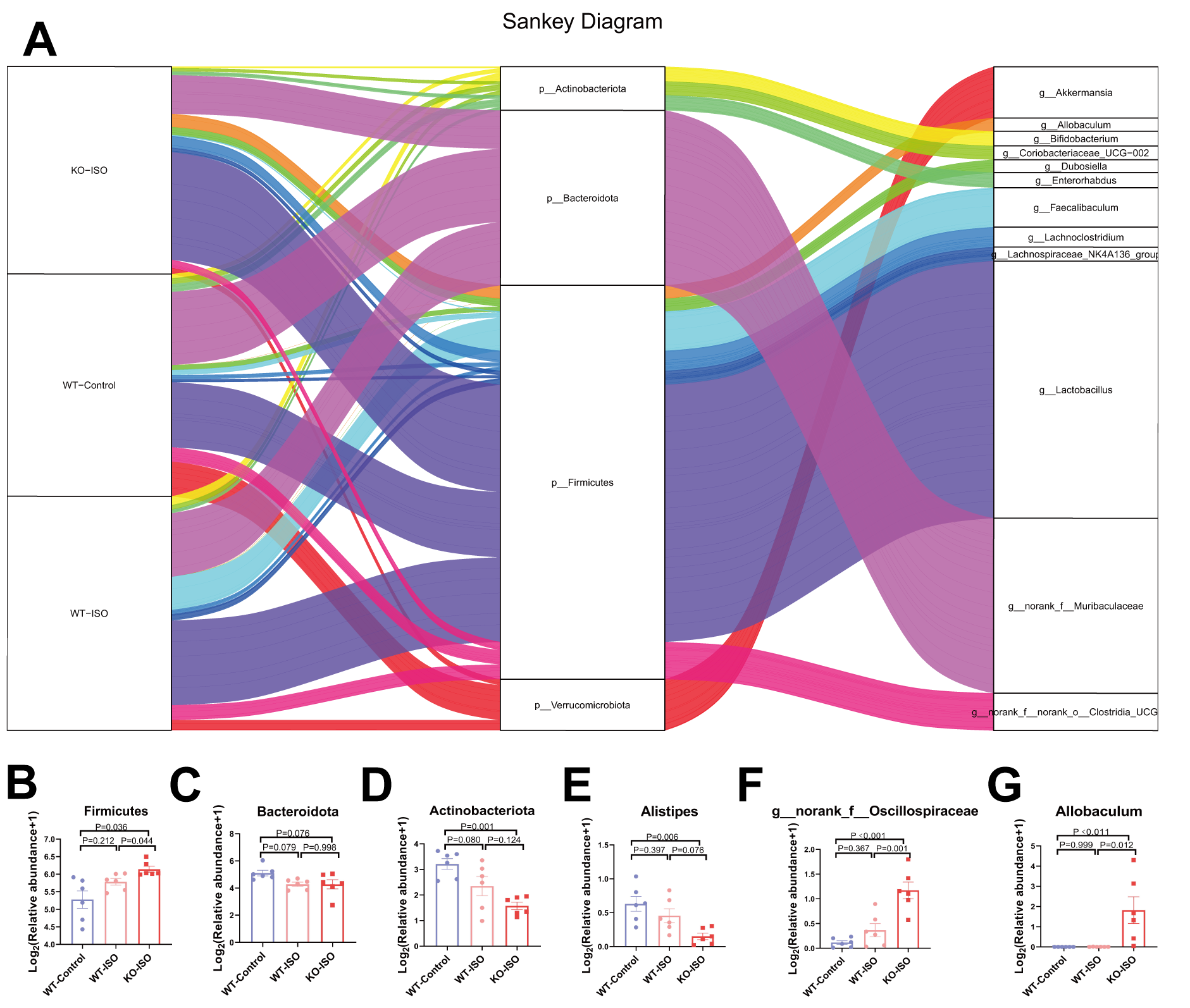

Supplement: SUPPLEMENTARY FIGURE S5 — Microbiome and metabolome alterations among WT-Control, WT-ISO and KO-ISO groups. (A) Sankey diagram was performed to analysis of species composition at the phylum and genus level. Relative abundance of (B) Firmicutes, (C) Bacteroidota and (D) Actinobacteriota at phylum level. Relative bundance of (E) Alistipes (F) g_norank_f__Oscillospiraceae and (G) Allobaculum at genus level. n = 6 mice per group. [file Image_5.TIF]

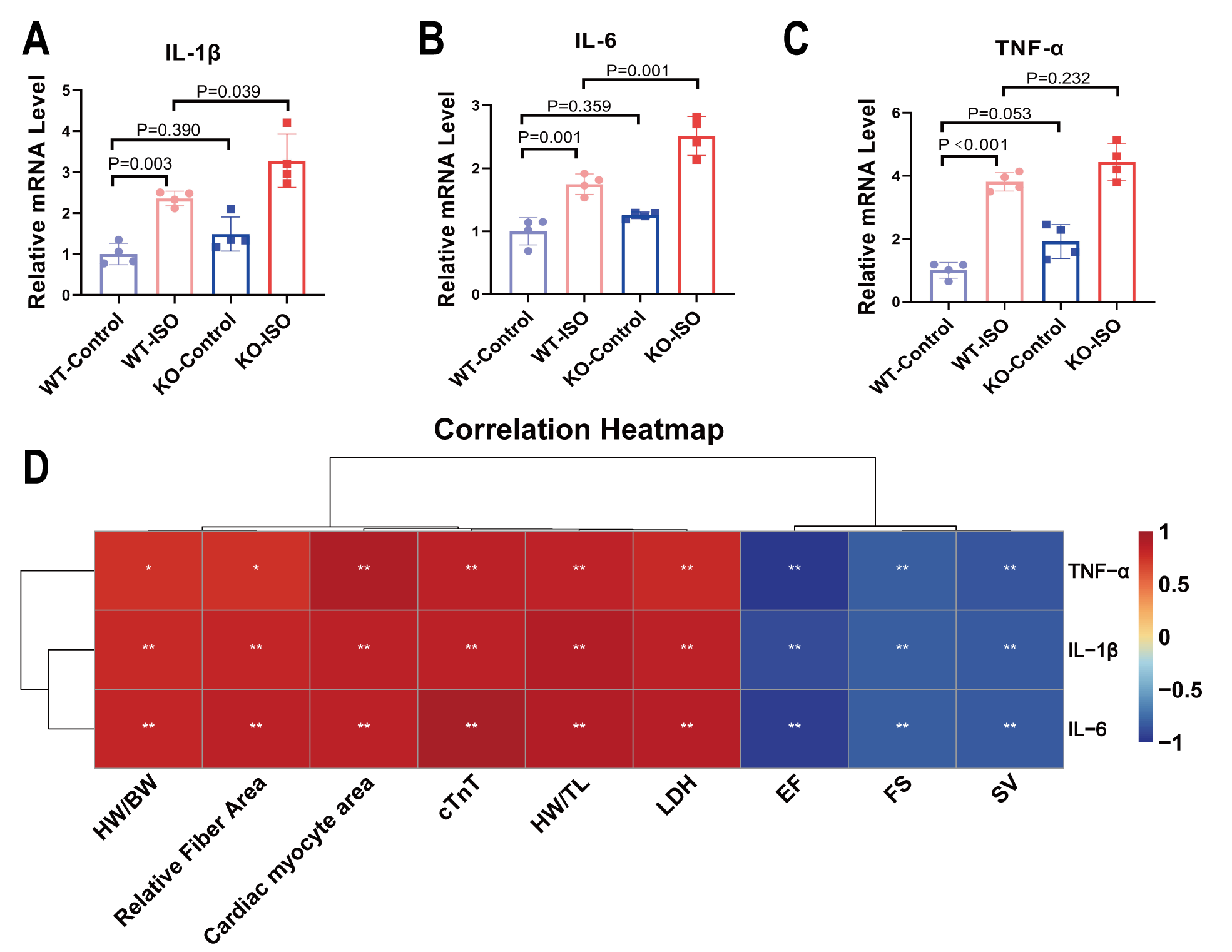

Supplement: SUPPLEMENTARY FIGURE S6 — Sigmar1-/- exacerbated ISO-induced elevated expression of cardiac inflammatory factors. The mRNA levels of (A) IL-1β, (B) IL-6 and (C) TNF-α among the four groups. (D) Spearman correlation analysis for 3 inflammatory factors and 9 cardiac-related indices. [file Image_6.TIF]
